# A Novel Sensitive Luminescence Probe Microspheres for Rapid and Efficient Detection of τ-Fluvalinate in Taihu Lake

**DOI:** 10.1038/srep46635

**Published:** 2017-05-09

**Authors:** Jixiang Wang, Yunyun Wang, Hao Qiu, Lin Sun, Xiaohui Dai, Jianming Pan, Yongsheng Yan

**Affiliations:** 1 School of Chemistry and Chemical Engineering, Jiangsu University, Zhenjiang 212013, People’s Republic of China; 2 Institute of Green Chemistry and Chemical Technology, Jiangsu University, Zhenjiang 212013, People’s Republic of China

## Abstract

Fluorescent molecularly imprinted polymers have shown great promise in biological or chemical separations and detection, due to their high stability, selectivity and sensitivity. In this work, fluorescent molecularly imprinted microsphere was synthesized via precipitation polymerization, which could separate efficiently and rapidly detect τ-fluvalinate (a toxic insecticide) in water samples, was reported. The fluorescent imprinted sensor showed excellent stability, outstanding selectivity and the limit of detection low to 12.14 nM, good regeneration ability which still kept good sensitivity after 8 cycling experiments and fluorescence quenching mechanism was illustrated in details. In addition, the fluorescent sensor was further used to detect τ-fluvalinate in real samples from Taihu Lake. Despite the relatively complex components of the environment water, the fluorescent imprinted microspheres sitll showed good recovery, clearly demonstrating the potental value of this smart sensor nanomaterial in environment monitoring.

Molecular imprinting, as a versatile and well-established technique, which is widely used for synthesizing molecularly imprinted polymers (MIPs) with specific molecular recognition properties, has attracted much attention in recent years^1^,^2^. The synthetic program is based on the copolymerization of functional monomers and cross-linkers to form complexes with the template molecules, after the removal of the template, the special tailor-made binding sites that are complementary in size shape and functionality to template are obtained^3^. Due to the desired predetermination, high specific selectivity and practicability, MIPs have been widely used in various significant applications, such as chemical sensor^4^, solid phase extraction^5^, artificial antibodies^6^, stationary phases in chromatography^7^, extraction and separation^8^,^9^, catalysis^10^,^11^,^12^, drug delivery and controlled release^13^,^14^,^15^ and so on. However, the bulk MIPs exhibit high selective recognition but have low binding kinetics, poor binding capacity, and shortcomings of incomplete template, attributing to the high difficult of the extraction of template molecules embedded inside the thick polymer network^16^. To overcome those problems, surface molecular imprinting technique has been developed and explored in recent years, which fabricated MIPs film at the surface of a solid support^17^,^18^,^19^. Through this unique way, the core–shell structural surface imprinted polymers are used efficiently to improve the binding kinetics, binding capacity, and site accessibility to the target molecules.

The central part of a chemical sensor is the recognition element in order to assess the binding events directly with a sensitive analytic procedure, for example fluorescence detection technique. Fluorescence detection based on fluorescent sensors is the most valuable method utilized in sensing thanks to its high sensitivity, simplicity, and diversity^20^,^21^,^22^,^23^. In so far, fluorescence detection is the superior detection means in the field of sensing technology attributing to several well-established advantages. The fluorescence chemical sensors and biosensors have been a hot topic on chemical analysis, biochemistry and medical science. For example, Feng and co-workers proposed fluorescence sensor for specific recognition and direct fluorescent quantification of perfluorooctane sulfonate^24^. Oh *et al*. reported fluorescent boronate-modified polyacrylonitrile nanoparticles of 50 nm diameter were fabricated for use as a selective H_2_O_2_ sensor^25^. Wu and co-workers proposed to prepare hydrophilic and peptide-functionalized upconversion fluorescent nanoparticles, which were used in the design of a biosensor for the sensitive and selective determination of human immunodeficiency virus antibodies in human serum^26^. In our group, Gao and co-workers have prepared one molecularly imprinted polymer microspheres for ultra-trace nonfluorescent cyhalothrin in honey^27^. Wei *et al*. used octadecyl-4-vinylbenzyl-dimethyl-ammonium chloride as a surfactant to transfer aqueous CdTe quantum dots for the recognition of cyhalothrin^28^. Many papers have already reported that some inorganic ions and small biomolecules could be detected by molecular imprinted technique based on the fluorescence quenching. Such as, Zhou and co-workers Synthesized fluorescence molecularly imprinted polymers as sensor for highly sensitive detection of dibutyl phthalate from tap water samples^29^. Panagiotopoulou *et al*. prepared fluorescent molecularly imprinted polymers as plastic antibodies for selective labeling and imaging of hyaluronan and sialic acid on fixed and living cells^30^. Sellergren’s groups prepared sialic acid-imprinted core–shell nanoparticles equipped with nitrobenzoxadiazole (NBD) fluorescent reporter groups allowing environmentally sensitive fluorescence detection at convenient excitation and emission wavelengths^31^. Ming and co-workers synthesized magnetic fluorescence molecularly imprinted polymers for detection of trace 17 beta-estradiol in environment water^32^. In a word, the high sensitivity of fluorescence detection technique combines with the advantages of highly selective MIPs can analyze trace substances in real samples and reduce the limit of detection.

Synthetic pyrethroid, whose structure was similar to natural chrysanthemum ester, have been widely used in agriculture and the control of insects in household application^33^,^34^. As a kind of synthetic pyrethroid, τ-fluvalinate (FL) disturbed consciousness and seizures by affecting the central nervous system of insects^35^,^36^. However, some researches indicated that it has endocrine-disrupting effects^37^. As far as we known, τ-fluvalinate can exist in the soil and surface of the crop over 13 days, then will seep into the soil and underground water^38^,^39^. Therefore, people and animals, especially fish and shrimp, are in danger while pesticides are producing and using, once diffused by insecticides, pesticides would contaminate agricultural products and water in the environment^40^,^41^,^42^. Hence, it urgently needs one kind of rapid, steady and efficient method to detect residual τ-fluvalinate, so as to protect human away from the contaminated environment. To date, several efficient methods for detecting synthetic pyrethroid have been widely applied, including gas chromatography^43^,^44^, high-performance liquid chromatography^45^, and thin-layer chromatography^46^,^47^. However, these methods suffer from high cost, long analysis time intervals, low selectivity, and possible production of secondary pollutants. Comparing with those, fluorescence chemical sensors or biosensors can make the detection simpler, more rapid and even can reduce the cost. Therefore, the toxic characteristics and highly hazardous of τ-fluvalinate require the development of simple, fast, sensitive, and accurate analytical methods for its detection and quantification.

In this work, we prepare fluorescent imprinted polymers as a sensor via precipitation polymerization based on SiO_2_, which were used for the sensitive and selective determination of τ-fluvalinate. To check the efficiency of this new sensor nanomaterial for detection of τ-fluvalinate, we chose the Taihu Lake water as a real sample. To the best of our knowledge, this molecularly imprinted fluorescent nanomaterial is the first example of a thin-film core-shell nanostructure that could be used as a fluorescent sensor for τ-fluvalinate in real water sample. Therefore, besides the immediate advantage in selective detection of pollutants from environmental waters, the efficient ability for pesticide detection demonstrated in this is promising in the context of agricultural products and food safety analysis.

## Experiment

### Reagents and Chemicals

All chemicals were of analytical grade reagents. Tetraethyl orthosilicate (TEOS), ammonium hydroxide, 3-Trimethylolpropane trimethacrylate (TRIM) Acrylamide (AM) and 3-(methacryloyloxy)propyltrimethoxysilane (KH570), were all purchased from Aladdin Chemistry Co. Ltd. (Shanghai, China). Acetonitrile, ethyl alcohol, fluorescein, allyl bromide, hydroquinone, iodine, N,N-Dimethylformamide (DMF), potassium carbonate (K_2_CO_3_), trichloromethane, silica gel, methyl alcohol and acetic acid were all obtained from Sinopharm Chemical Reagent Co., Ltd. (Shanghai, China). All the pyrethroid pesticides, including fenvalerate (FE), beta-cyfluthrin (BC), λ-Cyhalothrin (LC), bifenthrin (BI) and τ-fluvalinate (FL) were purchased from Yingtianyi standard sample company (Beijing, China). Doubly distilled water was used for cleaning processes. The chemical structures of pyrethroid were shown in Fig S1.

### Instruments

Fourier transform infrared (FTIR) spectra (4000–400 cm^−1^) were tested using KBr pellet in a Nicolet NEXUS-470 apparatus (U.S.A). The scanning electron microscope (SEM, JEOL, JSM-7001F) and transmission electron microscope (TEM, JEOL, JEM-2100) were utilized to observe the morphologies of SiO_2_@FL-FMIPs and SiO_2_@FNIPs. Fluorescence experiments were analyzed by Cary Eclipse fluorescence spectrophotometer (Varian, USA). TCS SP5 II confocal laser microscopy (Leica, Germany) was employed to observe the fluorescence intensity of SiO_2_@FL-FMIPs samples with a 488 nm solid state laser light source. Transient fluorescence spectra were recorded on a QuantaMaster™ 40 Spectrofluorometer (Photon Technology International, U.S.A). Thermal gravity analysis (TGA) of SiO_2_@BC-FMIP samples was performed using Diamond TG/DTA instruments (Perkin-Elmer, U.S.A).

### Preparation of Allyl fluorescein

Allyl fluorescein as a kind of fluorochrome was chosen with a simple and convenient method, which provided group of vinyl to reacted with cross-linking agent and monomer and form the polymer layer^48^. Many works use it to detect target through the fluorescence changes^49^,^50^. Figure S2 showed the synthesizing chemical equation of the allyl fluorescein. The concrete method as following: first, fluorescein (6.0 mmol), allyl bromide (20.0 mmol), K_2_CO_3_ (36.0 mmol), hydroquinone and iodine (trace) was dissolved into 60 ml of dry DMF in the dark under a nitrogen atmosphere for 25 h at 71 °C. The primary product was obtained under reduced pressure. Then, we utilized column chromatographic to purify for primary product and gain pure allyl fluorescein (yield, 63.3%).

### Preparation of SiO_2_ and SiO_2_-KH570

Silicon dioxide as a sort of substrate material has been widely applied into polymer polymerization to improve controllability of morphology and biocompatibility, because it does not only have a good performance of optical transparency and chemical inertness but also the particle surfaces can be modified readily with various functional group via commercially available agents.

In this case, SiO_2_ spheres were prepared with stöver process^51^,^52^. In brief, NH_3_·H_2_O (0.2 mL), ethanol (2.5 mL) and double distilled water (2.5 mL) were added successively into a round-bottomed flask with a mechanical stirring at room temperature for 0.5 h. Then, TEOS (0.2 mL) was dropped into the flask and the mixture was stirred at room temperature for 24 h. At last, SiO_2_ particle was obtained with a speed of 1000 rpm/min via centrifuge and dried in vacuum oven at 60 °C overnight.

To form a polymer layer on the surface of SiO_2_, SiO_2_ was modified with double bond which provided active group. Typically, first, 200 mg of SiO_2_ were dispersed into 60 mL of ethyl alcohol for 1 h. Then, 2 mL of KH570 were dropped at 40 °C for 24 h, The SiO_2_-KH570 particles were separated from ethyl alcohol via centrifuge with a speed of 1000 rpm/min and dried in vacuum oven at 60 °C overnight.

### Preparation of SiO2@FL-FMIPs and SiO2@FNIPs

**Figure 1:**
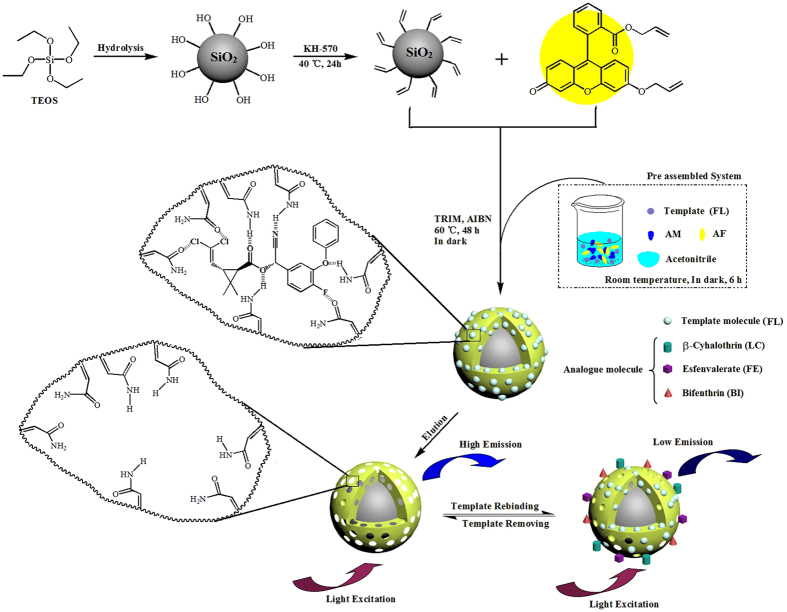
Schematic illustration for the preparation of SiO_2_@FL-FMIPs.

SiO_2_@FL-FMIPs were synthesized via precipitation polymerization and the synthetic process of SiO_2_@FL-FMIPs was shown in Fig. 1. Before the polymerization, we need to preassemble functional monomer, assistant functional monomer and target monomer. Therefore, AM (0.4 mmol), allyl fluorescein (0.01 mmol) and FL (0.1 mmol) was added to a one-neck round-bottom flask (100 mL) with 60 mL of acetonitrile at room temperature for 6 h. 200 mg of obtained SiO_2_-KH570 was uniformly dispersed into above-mentioned round-bottom flask. Then, TRIM (0.3 mL) was added successively into the mixture under a nitrogen atmosphere for 0.5 h. The flask was sealed and heated at 60 °C for 24 h with a magnetic stir at a stirring rate of 300 rpm. Function monomers and target monomer (τ-fluvalinate) are copolymerized with cross linkers in the desired surface molecule imprinted polymers. At last, the polymers were separated by centrifugation and washed repeatedly with ethyl alcohol and distilled water until that supernatant liquid was transparent, then dried at 40 °C under vacuum overnight.

Of course, the synthetic process of SiO_2_@FNIPs was similar to SiO_2_@FL-FMIPs except that template molecule was omitted.

### Fluorescence detection of SiO_2_@FL-FMIPs and SiO_2_@FNIPs

To obverse intuitively fluorescence detection of synthetic senor, the fluorescence spectrophotometer was used to detect fluorescence intensity variation of different materials and all the luminescence experiments were measured at room temperature. In luminescence experiments, the excitation wavelength and emission wavelength of synthetic senor were 488 nm and 525 nm, respectively.

To incarnate preciseness and veracity of experiments, some preparatory works need be completed before the spectrum measurement. 10 mg of SiO_2_@FL-FMIPs were dispersed in 50 mL of alcohol solution. Then, 5.0 mL of the SiO_2_@FL-FMIPs solution were taken out and added into FL solution which concentration ranges were 0–2.0 μM, respectively. The solution under measurement was shocked uniformly and detected the fluorescence intensity. When the quencher (FL) concentration increased to 160 nM, the quenching efficiency of SiO_2_@FL-FMIPs was maximum. We found that when the concentration of SiO_2_@FL-FMIPs was 10 mg, it showed an excellent quenching rate and meanwhile the fluorescence intensity (a.u.) will not beyond detection scope. Therefore, in our system 10 mg of SiO_2_@FL-FMIPs is the experiment optimization in fluorescence detection. At last, the fluorescence quenching efficiency (F_0_/F-1) of SiO_2_@FL-FMIPs with FL were calculated by Stern-Volmer equation. and a linear relationship of concentration (nM) and (F_0_/F-1) could be obtained by fitting. All the study of SiO_2_@FNIPs was performed under the identical conditions.

## Results and Discussion

### Characterization of SiO_2_@FL-FMIPs and SiO_2_@FNIPs

The FT-IR spectroscopy of SiO_2_ (a), SiO_2_@FL-FMIPs (b), SiO_2_@FNIPs (c) was shown in Fig. S3. In Fig. S3(a), the peaks of 803 cm^−1^ and 473 cm^−1^ were the characteristic signals of Si-O, which directed at bending vibration and symmetric stretching vibration, respectively. The peak of Si-O-Si asymmetric stretching was at 1106 cm^−1^. The above-mentioned data indicated that silicon dioxide was successfully prepared with stöver process. The curves (b) & (c) were FT-IR spectroscopy of SiO_2_@FL-FMIPs and SiO_2_@FNIPs, respectively. Compared with pure silicon dioxide, several peaks were increased. The aliphatic stretching of the C-H bond was at 2977 cm^−1^. The peak of 955 cm^−1^ was the bending vibration of Si-O-C. The peak illustrated the existence of KH570. The characteristic peak of perfluoroalkyl (-CF_3_) appeared at 1403 cm^−1^. It represented the analytes (FL) was successfully polymerized in imprinted layer. Moreover, the strong peak of 1733 cm^−1^ was the characteristic peak of -COO- and it was contained in both KH570 and FL. On the whole, the imprinted layer was successfully polymerized on the surface of SiO_2_.

The thermogravimetric analysis (TGA) of SiO_2,_ SiO_2_@FL-FMIPs and SiO_2_@FNIPs experiments were also carried out, with condition under nitrogen atmosphere up to 800 °C with heating rate of 10 °C·min^−1^. As shown as Fig. S4, the whole process of thermal weight loss could be divided into two stages roughly, from TGA curve of SiO_2_@FL-FMIPs and SiO_2_@FNIPs. The initial weight decrease was due to the loss of interior crystallization water. As the temperature range increased to 320–480 °C, the weight losses of SiO_2_@FL-FMIPs and SiO_2_@FNIPs reached to ~42.92% and ~42.57%, respectively. Which were corresponding to the decomposition temperature of the crosslinking agent and functional monomer. The similar TGA curve of SiO_2_@FNIPs was shown in Fig. S4. These result further demonstrated that the success of grafting molecularly imprinted polymers on SiO_2_. More importantly, the TGA results also indicated an almost identical polymer layer on SiO_2_@FL-FMIPs and its control one SiO_2_@FNIPs.

**Figure 2:**
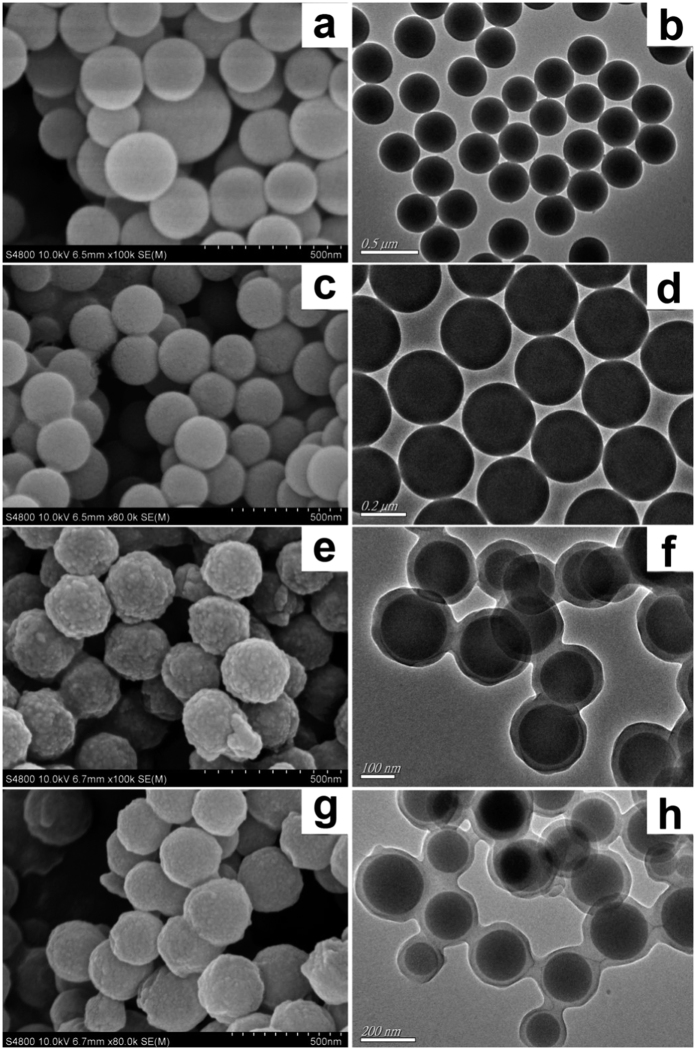
SEM images of SiO_2_ (**a**), SiO_2_-KH570 (**c**), SiO_2_@FL-FMIPs (**e**) and SiO_2_@FNIPs (**g**). TEM images of the SiO_2_ (**b**), SiO_2_-KH570 (**d**), SiO_2_@FL-FMIPs (**f**) and SiO_2_@FNIPs (**h**).

As shown as in Fig. 2, morphologies of SiO_2_ particle (a, b), SiO_2_-KH570 (c, d), SiO_2_@FL-FMIPs (e, f) and SiO_2_@FNIPs (g, h) were distinctly described via SEM and TEM technique. As shown in Fig. 2, the original SiO_2_ and SiO_2_-KH570 microspheres were both smooth and which the microspheres average size was ~300 nm. Moreover, the SEM and TEM images of SiO_2_-KH570 in Fig. 2(c,d), it was similar to pure SiO_2_ and the size was relatively symmetrical. Which was demonstrated although the surface of SiO_2_ was modified with KH570, morphologies of SiO_2_-KH570 was not altered. However, after surface imprinted polymerization, the diameter of the microspheres dramatically increases to ~350 nm, the morphologies and sizes of SiO_2_@FL-FMIPs/FNIPs were incarnated in Fig. 2(e & g,f & h). showing a 25 nm of molecularly imprinted layer on the SiO_2_ surfaces. Despite of the rough surface, the core-shell SiO_2_@FL-FMIPs still possessed well monodispersity and good approximately spherical morphology. The size distribution index was around 1.0694, according to our previous work^19^. Detailed calculation method was shown in the supplementary material. In a word, the SEM and TEM images proved that SiO_2_@FL-FMIPs with core-shell structure had been prepared via precipitation polymerization successfully. Further, confocal laser scanning microscope (CLSM) as a widespread imaging technology was used to observe the fluorescence images of SiO_2_@FL-FMIPs and SiO_2_@FNIPs. Before the measurement, excitation wavelength and emission wavelength were 488 nm and 525 nm, respectively. Apparently, as shown as in. SiO_2_@FL-FMIPs and SiO_2_@FNIPs both had green-yellow fluorescence. Compared with SiO_2_@FNIPs, the fluorescence of SiO_2_@FL-FMIPs was stronger, that is to say target monomer added could promote AF reacted with function monomer and cross-linking agent. In brief, CLSM measurement could indirectly illustrate that SiO_2_@FL-FMIPs and SiO_2_@FNIPs was synthesized successfully, the prepared material had good fluorescence and spherical morphology. As shown in Fig. S5(c′), the bright field image proves that allyl fluorescein is successfully grafted onto the surface molecular imprinting polymers.

X-ray photoelectron spectroscopy (XPS) wide scans and narrow scans were used to further analyze the imprinted layer composition of fluorescent imprinted sensors in Fig. S6. Figure S6(b) showed the peak of SiO_2_. Compared with pure SiO_2,_ the narrow scans for C1s peaks of SiO_2_-KH570 were in Fig. S6(c), it showed the peaks of C-O, C = C, C-C and indicated SiO_2_ had been successfully modified with KH570. The full XPS spectrum survey of SiO_2_@FL-FMIPs (Fig. S6(a) showed the presence of F, N element, together with narrow scans for F1s and N1s (Fig. S6(d and e)). As shown as in Fig. S6(d), it could be seen the peak of F-C, which was ascribed to template molecule (FL), and F-H, which was the hydrogen bond between template molecule (FL) and functional monomer (AM). In Fig. S6(e), the binding peaks of C-NH-C, N-C emerged, also demonstrated the presence of FL. Moreover, the peak of O = C-NH was ascribed to the functional monomer (AM). On the whole, XPS spectrum indicated the formation of imprinted layer on SiO_2_.

### Fluorescence detection performance

**Figure 3:**
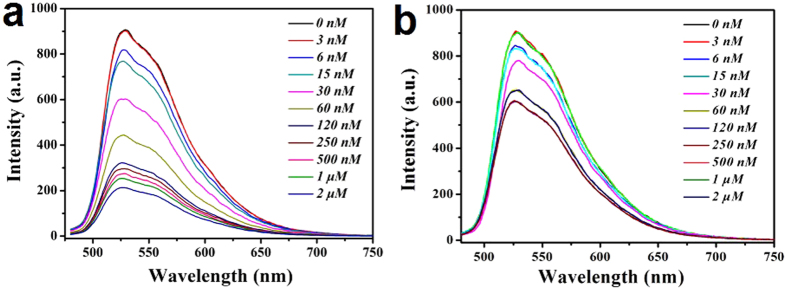
Response of SiO_2_@FL-FMIPs (**a**) and SiO_2_@FNIPs (**b**) to FL in the concentration range of 0–2.0 μM.

The fluorescence performance is important basis to evaluate the selective recognition and detection sensitivity of SiO_2_@FL-FMIPs and SiO_2_@FNIPs. The fluorescence intensity curves of SiO_2_@FL-FMIPs were shown in Fig. 3(a). We found that with the quencher (template molecules FL) concentration increasing, the fluorescence intensity (a.u.) was gradually decreased. In addition, the fluorescence intensity decreased obviously when the quencher concentration was at a range of 0-120 nM. In the same condition of quencher (FL) concentration, the fluorescence intensity curves in Fig. 3(b) were also found in a slight downward trend but not obvious. However, when the quencher concentration increased to above 120 nm, the quenching efficiency of SiO_2_@FL-FMIPs and SiO_2_@FNIPs both became inferior. This is understandable because the SiO_2_@FL-FMIPs formed specific FL binding sites during the imprinting process. In short, the fluorescence intensities could illustrated that the spatial adsorption sites could be incorporated into SiO_2_@FL-FMIPs matrix.

### Fluorescent detection kinetics

**Figure 4:**
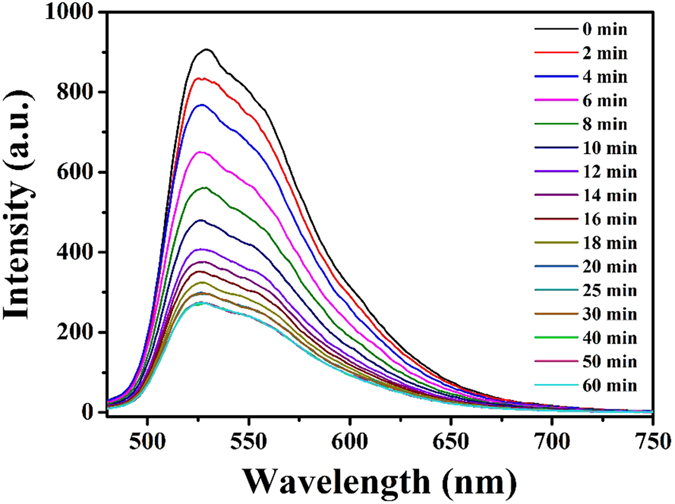
Dynamic fluorescent analysis detection time of SiO_2_@FL-FMIPs to FL (60 nM) within 60 min.

Compared with other detection methods, such as GC, HPLC and TLC, the fluorescent sensor-like analysis in this work was more simple and efficient. To verify the advantage of fast detection for FL, the dynamic binding performance experiment of was done and the concrete procedures were as following. 5 mL of the SiO_2_@FL-FMIPs solution was added into 5 mL of FL solution (60 nM, and the fluorescence intensity was tested in 90 minutes repeatedly. As shown as in Fig. 4, we could find the fluorescence intensity was decreasing as time goes on. Moreover, after incubation of the target molecules with SiO_2_@FL-FMIPs, the fluorescence quenching could rapidly reach to equilibrium within 12 min. In other word, fluorescence had been effectively quenched by the target molecule (FL). This result illustrated that the SiO_2_@FL-FMIPs microspheres showed excellent fluorescent detection kinetics due to the thin core-shell structure nanostructures. Taken together, all the findings above demonstrated that further demonstrated the fluorescent sensors could catch and recognize FL quickly, accurately and efficiently in actual samples.

### Selectivity determination

**Figure 5:**
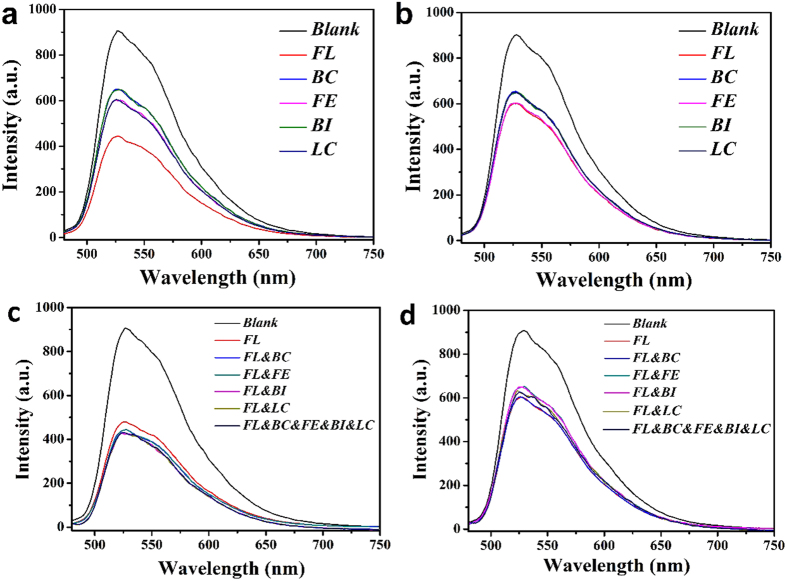
Fluorescence intensity curves of SiO_2_@FL-FMIPs (**a**) and SiO_2_@FNIPs (**b**) in different kinds of 60 nM pyrethroid. Fluorescence intensity curves of SiO_2_@FL-FMIPs (**c**) and SiO_2_@FNIPs (**d**) in different kinds of pyrethroid mixed solutions (60 nM for each pyrethroid).

The binding selectivity of the molecularly imprinted polymers is often determined by comparing the binding of the template with those of its analogs. To measure the selectivity of the SiO_2_@FL-FMIPs, we made a comparison between the potential interferences (three structurally related compounds, BC, FE, BI and LC) and FL. Further, selectivity determination experiment was required to measure the selectivity of the SiO_2_@FL-FMIPs. Specific procedures as following: same amount of SiO_2_@FL-FMIPs were added to 10 mL of ethanol solutions containing 60 nM of FL, BC, FE, BI and LC, respectively. The mixture was stirred for 0.5 h at room temperature, which was to adequately mix with quencher. In the end, the fluorescence intensity was measured via the fluorescence spectrophotometer. As shown in Fig. 5(a) and Fig. S7, it showed that the fluorescence intensity for FL was weaker than other potential interferences, this suggested that only FL could be efficiently recognized, exhibiting the excellent recognition ability of SiO_2_@FL-FMIPs. Furthermore, as shown in Fig. 5(b), there was almost no fluorescence quenching of SiO_2_@FNIPs towards all the quenchers (BC, FE, BI, LC and FL), probably due to the non-specific recognition sites of SiO_2_@FNIPs. These results clearly demonstrated the high selectivity of the SiO_2_@FL-FMIPs towards FL.

To further the investigation how the competitive analog affects the fluorescence quenching, and check the selectivity of SiO_2_@FL-FMIPs further in a complex environment. Quenching efficiency of SiO_2_@FL-FMIPs and SiO_2_@FNIPs by different kinds of 60 nM pyrethroid was shown in Fig. 5. The three competitive pesticides (BC, FE and BI) were added into LC solution to form mixed solutions, and the concentrations of FL and the competitive pyrethroid were all fixed at 60 nM. It was obviously seen that the proposed complex environment had almost no effect on the efficient detection of FL in Fig. 5(c). Although the mixed solution contained several structural analogs, the SiO_2_@FL-FMIPs could also efficiently detect the FL by fluorescence quenching as before. As also shown in Fig. 5(d), fluorescence quenching effect of SiO_2_@NIPs was still low. Therefore, the SiO_2_@FL-FMIPs definitely possessed excellent selectivity towards FL, and it put up great anti-interference performance for structural analogs. So the proposed complex environment had almost no effect on the detection of FL. This result also provided a possibility that the SiO_2_@FL-FMIPs imprinted fluorescent sensor nanomaterials could be used to identify FL from the complex real samples, for example, the environmental water.

### Fluorescence quenching mechanism

The transient fluorescence experiment of SiO_2_@FL-FMIPs and SiO_2_@FNIPs was measured to obtain F_0_ and the fluorescence curvy were displayed in Fig. S8, which illustrated the fluorescence lifetime curves. Fitted equation 1 is a single exponential decay equation between fluorescence lifetime (τ) and fluorescence intensity (F) of time (t):


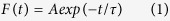


where A is amplitude, *τ* (ns) is the fluorescence lifetime.

As shown in Fig. S8, obvious fluorescence decay was found. The two decay curves were fitted by exponential function above (equation 1). The parameter of transient fluorescence spectrum was listed in the following Table S1.

In general, the fluorescence quenching process may occur in the interaction between the excited state molecules of fluorescent material and the quencher, and may also occur in the interaction between the ground state molecules of the fluorescent substance and the quencher. The former is called dynamic quenching, and the latter is called static quenching. In the process of dynamic quenching^53^,^54^, the excited state molecule of the fluorescent material returns to the ground state by colliding with the quencher molecule, loss of excitation energy by the mechanism of energy transfer or the mechanism of charge transfer. It can be seen that the efficiency of dynamic quenching is controlled by the lifetime of the excited state and the concentration of the quencher. The static quenching^55^ is characterized by the complex reaction between the quencher and the fluorescent substance in the ground state, the resulting complexes are usually non luminescent. To investigate the fluorescence quenching mechanism of SiO_2_@FL-FMIPs with FL, the Stern-Volmer equation (1) was transformed to another form, as follow:


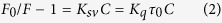


Where, *F_0_* is the initial fluorescence intensity without template molecules quenching agent. *F* is the fluorescence intensity with the concentration of template molecules quenching agent. *K_sv_* is quenching constant of Stern-Volmer, it is the ratio of the bimolecular quenching rate constant and the unimolecular decay rate constant, unit is L·mol^−1^. *C* is the concentration of template molecules quenching agent. *K_q_* is rate constant of bimolecular quenching process, unit is L·mol^−1^·s^−1^. *τ_0_* is lifetime without template molecules quenching agent.

The lifetimes are quite different and depend on whether the quenching agent exists or not. According to the difference of fluorescence lifetime in the presence of quencher and quenching nonentity (Table S1), In the case of dynamic quenching^56^, the presence of quencher shortens the fluorescence lifetime, *F*_*0*_*/F* = *τ*_*0*_*/τ*. Therefore, we can found another kind of representation of the Stern-Volmer equation (2):


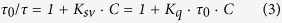


Where, *τ* is the lifetime with template molecules quenching agent and *τ_0_* is the lifetime without template molecules quenching agent.

According to eq (3), we known the relationship *K*_*sv*_* = K*_*q*_*·τ*_*0*_. Therefore, the rate constant K_q(MIPs)_ and K_q(NIPs)_ were calculated to be 6.648 × 10^9^ and 1.757 × 10^9^ L·mol^−1^*·*s^−1^ by eq (3), respectively. K_q_ is the rate constant of bimolecular quenching process, which is between quencher and fluorescent molecule, and K_q_ is between 1 × 10^9^ to 2.0 × 10^10^ L mol^−1^ s^−1^ for the dynamic quenching^57^. In this work, the K_q_ was never more than 2.0 × 10^10^ L·mol^−1^*·*s^−1^ and K_q(MIPs)_ (6.648 × 10^9^ L·mol^−1^*·*s^−1^) is more than K_q(NIPs)_ (1.757 × 10^9^ L·mol^−1^*·*s^−1^). Thus we deduced that the fluorescence quenching was a dynamic quenching process, and the SiO_2_@FL-FMIPs were more inclined to cause fluorescence quenching phenomenon than SiO_2_@FNIPs. Therefore, due to the existence of specific binding sites in the FL-imprinted thin core-shell structure microspheres, the SiO_2_@FL-FMIPs are more likely to occurring bimolecular dynamic collision and produce dynamic quenching as compared to SiO_2_@FNIPs.

Figure S9 showed the changes of fluorescence quenching efficiency *vs.* FL concentration (0–2.0 μM). There was a good linear relationship between fluorescence intensity and FL at the concentration range of 0–120 nM. The limit of detection (LOD) was calculated as 12.14 nM by equation of *LOD = 3·σ/S* (σ is the standard deviation of the blank signal and S is the slope of the linear calibration plot)^58^,^59^. According to the linear fitting curve of SiO_2_@FL-FMIPs and SiO_2_@NIPs at the concentration range of 0~120 nM, their quenching constant K_sv_ were calculated as 1.64 × 10^4^ and 3.1 × 10^3^* *L·mol^−1^, respectively. Thus, at the concentration range of 0~120 nM, the SiO_2_@FL-FMIPs could be used to quantitatively detect FL. Particularly worth mentioning is, as compared to previous work, we found that the SiO_2_@FL-FMIPs exhibited lower detection limit and wider detection range. (Table S2)

### Stability and regeneration performance

Fluorescence intensity stability was one important property for SiO_2_@FL-FMIPs to be applied into manufacture and it is necessary to study the fluorescence intensity stability of this material. Fluorescence stability was evaluated via measuring the fluorescence intensity of SiO_2_@FL-FMIPs every 5 min at room temperature. As shown in Fig. S10, there was no significant change on the fluorescence intensity of SiO_2_@FL-FMIPs after 13-times measurements within 60 min, which definitely demonstrated the fluorescence intensity stability of SiO_2_@FL-FMIPs. The results also demonstrate that the allyl fluorescein was effectively anchored into the network of SiO_2_@FL-FMIPs and the thin-film core-shell structure fluorescent microspheres had excellent fluorescence intensity stability.

The regeneration performance of SiO_2_@FL-FMIPs were further investigated by measuring the capacity of detection and adsorption of FL through a cyclic method. As shown in Fig. S11, the fluorescence intensity histogram of the eight experiments, where we could find that the fluorescence intensity of previous seven times experiments without obvious decrease of signal intensity, and after the sixth one, the fluorescence intensity started to decline a little. The SiO_2_@FL-FMIPs could be recycled at least seven times with a good selectivity determination and absolutely adapt with the requirement of manufacture reproducibility. Figure S12 shown the response curve of SiO_2_@FL-FMIPs to FL in the concentration range of 0–60 nM by seventh recycled materials. It can be seen that material still can detect FL by fluorescence quenching after seven regeneration. Therefore, the SiO_2_@FL-FMIPs could exhibit continuous superior performance in a cyclic FL fluorescence detection.

### Real water sample analysis

To assess the applicability of SiO_2_@FL-FMIPs in real water samples, we collected MiniQ water, tap water and the Taihu Lake water as different FL-containing samples for fluorescence analysis. Taihu Lake is located in the Yangtze River delta, where the area has the developed agriculture and fishery. Therefore, it is necessary for detection and control of synthetic pyrethroids to preserve human and aquatic organisms from danger. For filtered real water samples, the Taihu Lake water in Wuxi, Suzhou and Huzhou stream segment were filtrated and centrifuged to remove the insoluble impurities prior to use, and these Taihu Lake water samples were marked as Taihu Lake water sample 1, 2, 3, respectively. Afterwards, real water samples of different concentration ranges (0–500 nM) were prepared. Then 5.0 mL of the SiO_2_@FL-FMIPs solutions, as mentioned above, was added into various concentration of real water sample with thorough shake. To reflect the veracity and preciseness of experiment, the uniform procedure was repeated three times, respectively and average value was gained. As shown in Table 1, the SiO_2_@FL-FMIPs sensor nanomaterials showed excellent recovery in the linear concentration range discussed above (0–120 nM), regardless of MiniQ water, tap water or the real Taihu Lake water. The recovery of FL-containing water samples detected by using HPLC was display in Table S3, compare with instrument detection, this fluorescence sensor also has good recovery. Merely, the recovery became poorer in the case of the concentration above 120 nM (beyond the linear range). Nevertheless, the SiO_2_@FL-FMIPs sensor nanomaterials still exhibited almost equivalent recovery in the Taihu Lake water as compared to that in MiniQ water and tap water. Taken together, these results clearly demonstrated the potential of our thin-film core-shell molecularly imprinted fluorescent microspheres as a rapid and efficient sensor to detect FL in realistic environment water.

## Conclusions

In summary, a thin core-shell FL-imprinted fluorescent polymeric microspheres was successfully synthesized and used as fluorescent sensor in environment monitoring. The resultant nanosensor was not only proved to be a novel and sensitive luminescence probe for optical recognition of FL, but also expanded the potential application of MIPs. The practical fluorescent detection results illustrated that the LC-imprinted fluorescent nanosensor exhibited excellent sensitivity, selectivity, reusability and stability, and indicated the fluorescence quenching mechanism in detail. Moreover, the thin core-shell FL-imprinted fluorescent microspheres could be used for efficient detection of real water sample from Taihu Lake water, thus clearly indicating its great potential in the selective detection of pollutants from real environmental waters.

## Additional Information

**How to cite this article:** Wang, J. *et al*. A Novel Sensitive Luminescence Probe Microspheres for Rapid and Efficient Detection of τ-Fluvalinate in Taihu Lake. *Sci. Rep.* **7**, 46635; doi: 10.1038/srep46635 (2017).

**Publisher's note:** Springer Nature remains neutral with regard to jurisdictional claims in published maps and institutional affiliations.

## Supplementary Material

Supplementary Information

## Figures and Tables

**Table 1:** The recovery of FL-containing water samples detected by using the thin core-shell molecularly imprinted fluorescent nanosensor.

Samples	Test	FL added (nM)	FL detected* (nM)	Recovery (%)
MiniQ water	1	0	0.2 ± 0.03	—
	2	30	27.7 ± 1.6	92.2 ± 5.6
	3	60	57.5 ± 1.3	95.8 ± 2.3
	4	120	123.3 ± 6.9	102.8 ± 5.6
	5	500	425.1 ± 39.3	85.0 ± 9.3
Tap water	1	0	0.1 ± 0.04	—
	2	30	34.7 ± 3.6	115.5 ± 10.3
	3	60	62.7 ± 3.6	104.4 ± 5.7
	4	120	124.6 ± 2.1	103.8 ± 1.6
	5	500	405.7 ± 45.6	81.1 ± 11.2
Taihu Lake water 1	1	0	0.2 ± 0.04	—
	2	30	33.3 ± 1.8	111.1 ± 5.3
	3	60	63.3 ± 1.7	105.5 ± 2.7
	4	120	128.6 ± 6.3	107.2 ± 4.7
	5	500	400.3 ± 22.5	80.1 ± 5.5
Taihu Lake water 2	1	0	0.2 ± 0.1	—
	2	30	34.2 ± 3.2	113.9 ± 9.4
	3	60	61.5 ± 5.3	101.7 ± 8.7
	4	120	128.6 ± 7.2	107.2 ± 0.9
	5	500	417.8 ± 33.3	83.4 ± 7.9
Taihu Lake water 3	1	0	0.3 ± 0.1	—
	2	30	33.1 ± 2.1	110.2 ± 6.3
	3	60	62.3 ± 4.9	103.8 ± 7.8
	4	120	124.9 ± 5.9	104.1 ± 3.9
	5	500	410.2 ± 57.8	82.1 ± 14.1

^*^Average of three measurements.
