# Supplementary material for: A Novel Sensitive Luminescence Probe Microspheres for Rapid and Efficient Detection of τ-Fluvalinate in Taihu Lake
Source: Sci Rep. 2017 May 9;7:46635. doi: 10.1038/srep46635 (PMC5423034; doi:10.1038/srep46635)
Supplement: Supplementary Information [file srep46635-s1.doc]

*Supplementary Information*

**A Novel Sensitive Luminescence Probe** **Nanospheres for Rapid and Efficient Detection of τ-Fluvalinate in Taihu Lake**

*Jixiang Wang^1^, Yunyun Wang^1^, Hao Qiu^1^, Lin Sun^1^, Xiaohui Dai^1,2^, Jianming Pan^1,2^, Yongsheng Yan1,2[[1]](#footnote-2)**

*^1^ School of Chemistry and Chemical Engineering, Jiangsu University, Zhenjiang 212013, People’s Republic of China*

*^2^ Institute of Green Chemistry and Chemical Technology,* *Jiangsu University, Zhenjiang 212013, People’s Republic of China*

Figure S1. The chemical structures of all pyrethroids.


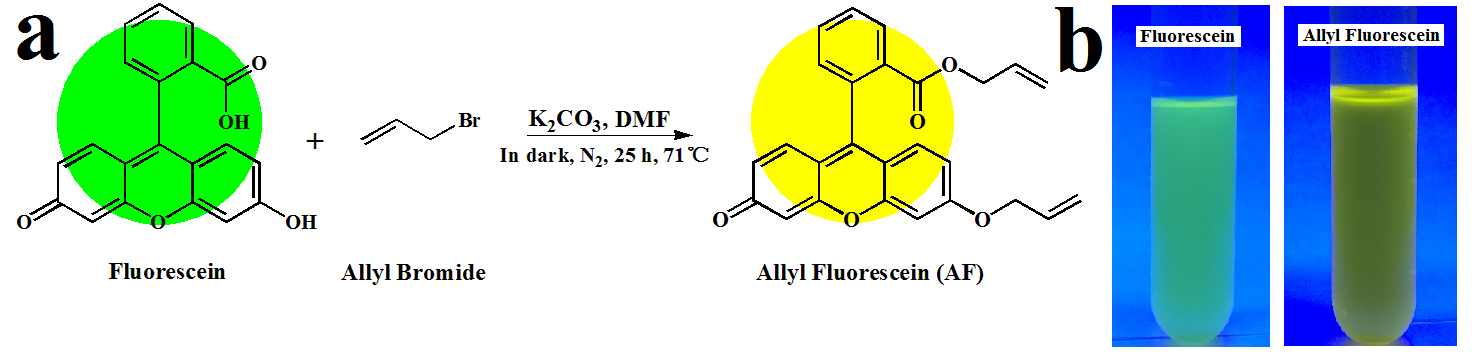


Figure S2. Synthetic scheme of Allyl fluorescein (AF)


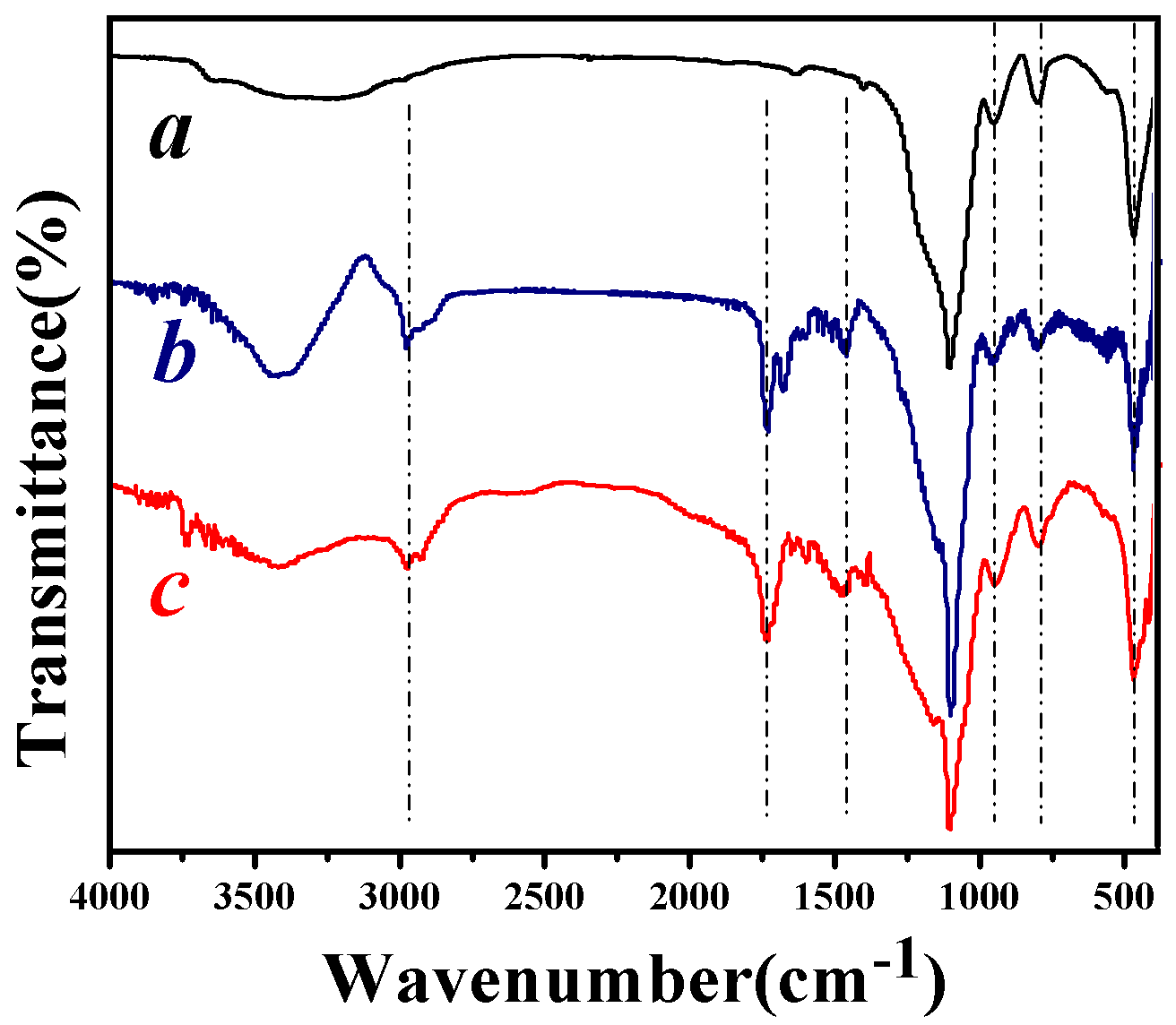


Figure S3. FT-IR spectra of SiO_2_ (a), SiO_2_@FL-FMIPs (b) and SiO_2_@FNIPs (c).


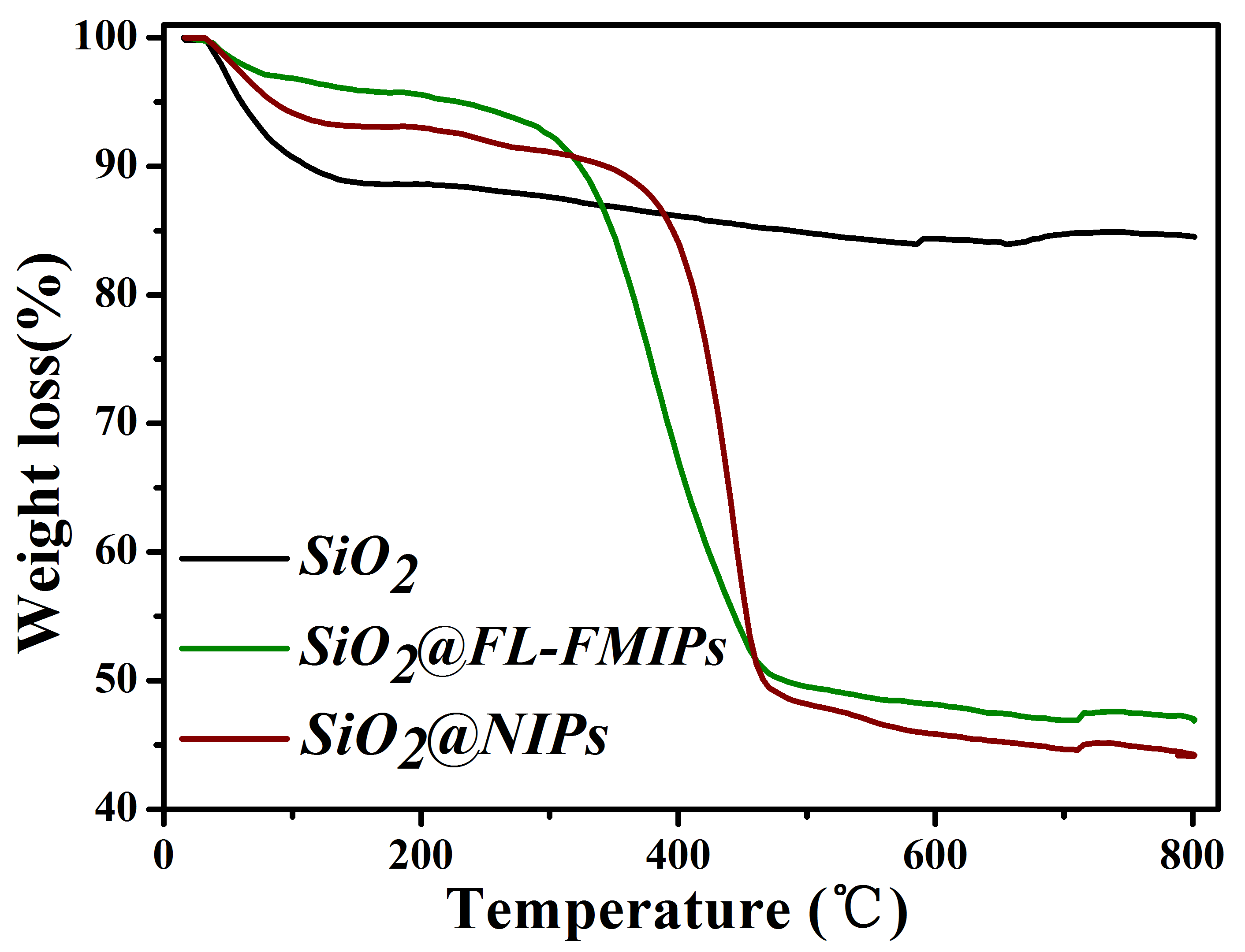


Figure S4. TGA curves of SiO_2_, SiO_2_@FL-FMIPs and SiO_2_@FNIPs at a heating rate of 10℃·min^-1^ from room temperature to 800℃ under N_2_ atmosphere.

The morphologies, particle sizes, and size distributions of the FMIP/FNIP microspheres were characterized with a scanning electron microscope. All SEM size data reflect the averages about 100 particles (they represent all the particles in one representative area in the SEM image), which are calculated by the following formulas:

*k k* *k k*

*D*_n_ = ∑ *n_i_D_i_*/ ∑*n_i_* ; *D*_w_ = ∑*n_i_D_i_*^4^/∑*n_i_D_i_*^3^ ; *U* = *D*_w_*/D*_n_

*i*=1 *i*=1 *i*=1 *i*=1

where *D*_n_ is the number-average diameter, *D*_w_ the weight-average diameter, *k* the total number of the measured particles, *D_i_* the diameter of the measured microspheres, *n_i_* the number of the microspheres with a diameter *D_i_*, and *U* the size distribution index.


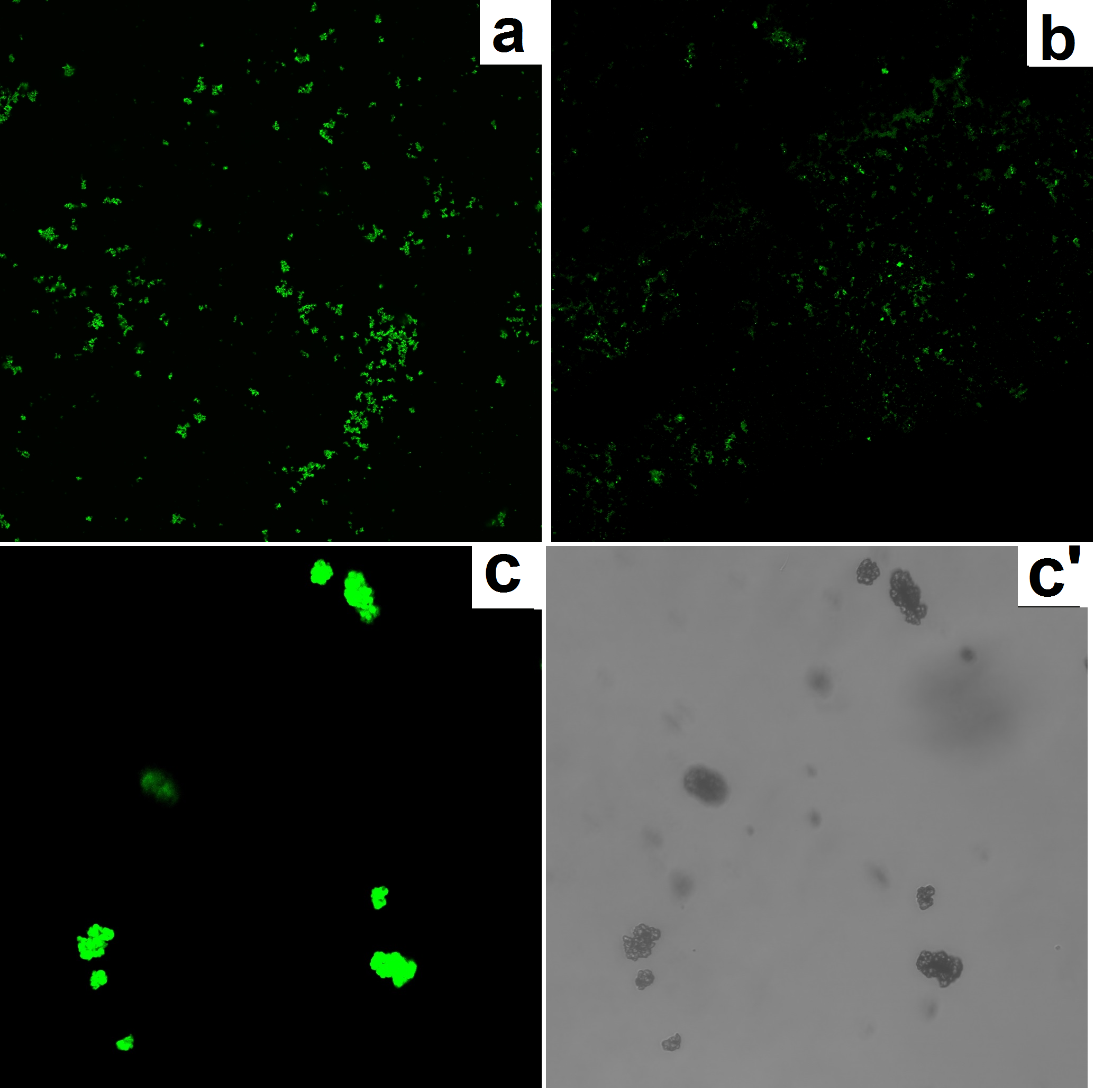


Figure S5. CLSM images and bright field image of SiO_2_@FL-FMIPs (a,c), SiO_2_@FNIPs (b) and SiO_2_@FL-FMIPs bright field image(c’)


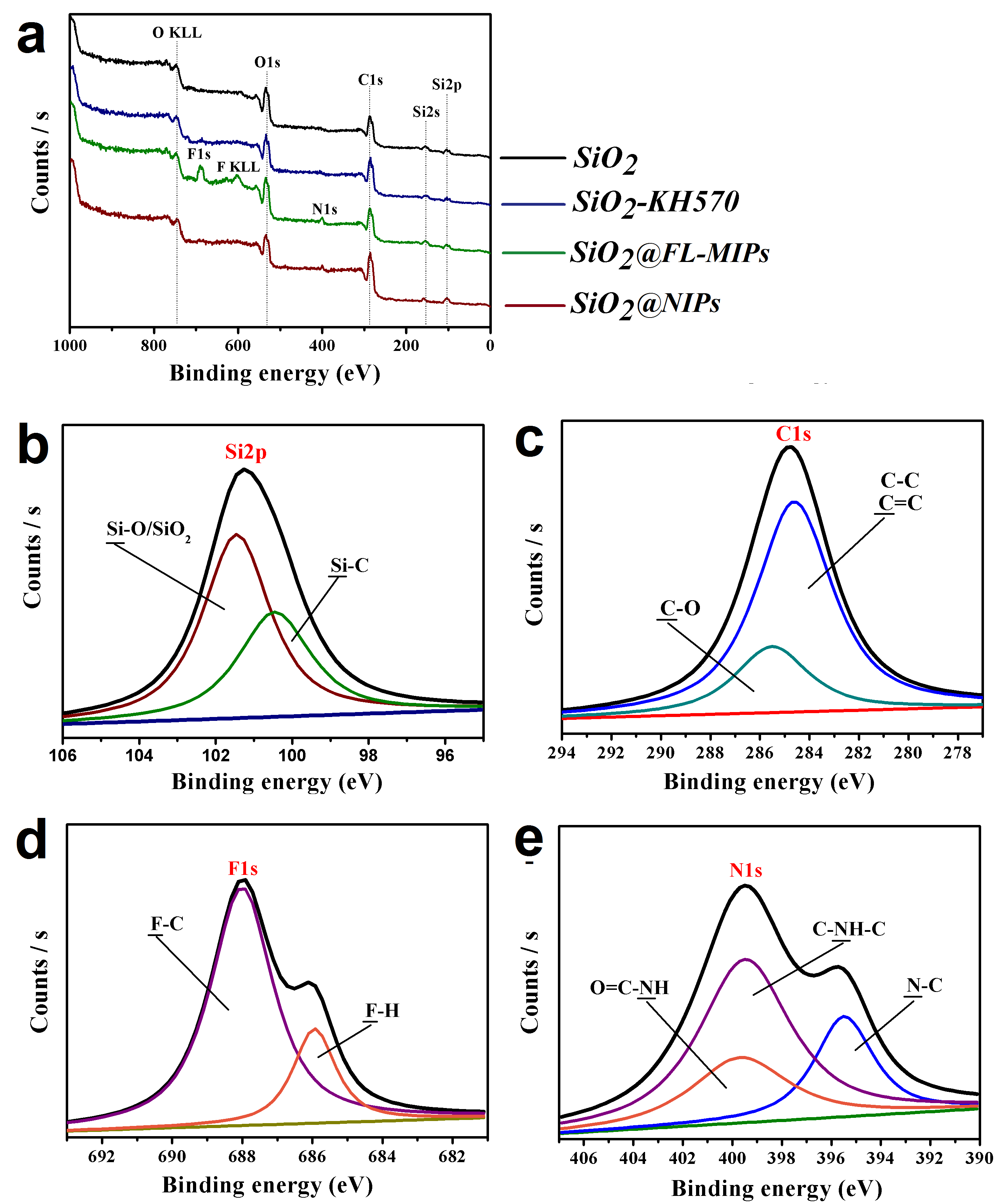


Figure S6. a) XPS wide scans of pure SiO_2_, SiO_2_-KH570, SiO_2_@FL-FMIPs, SiO_2_@FNIPs and narrow scans for b) Si2p, c) C1s, d) F1s, e) N1s peaks


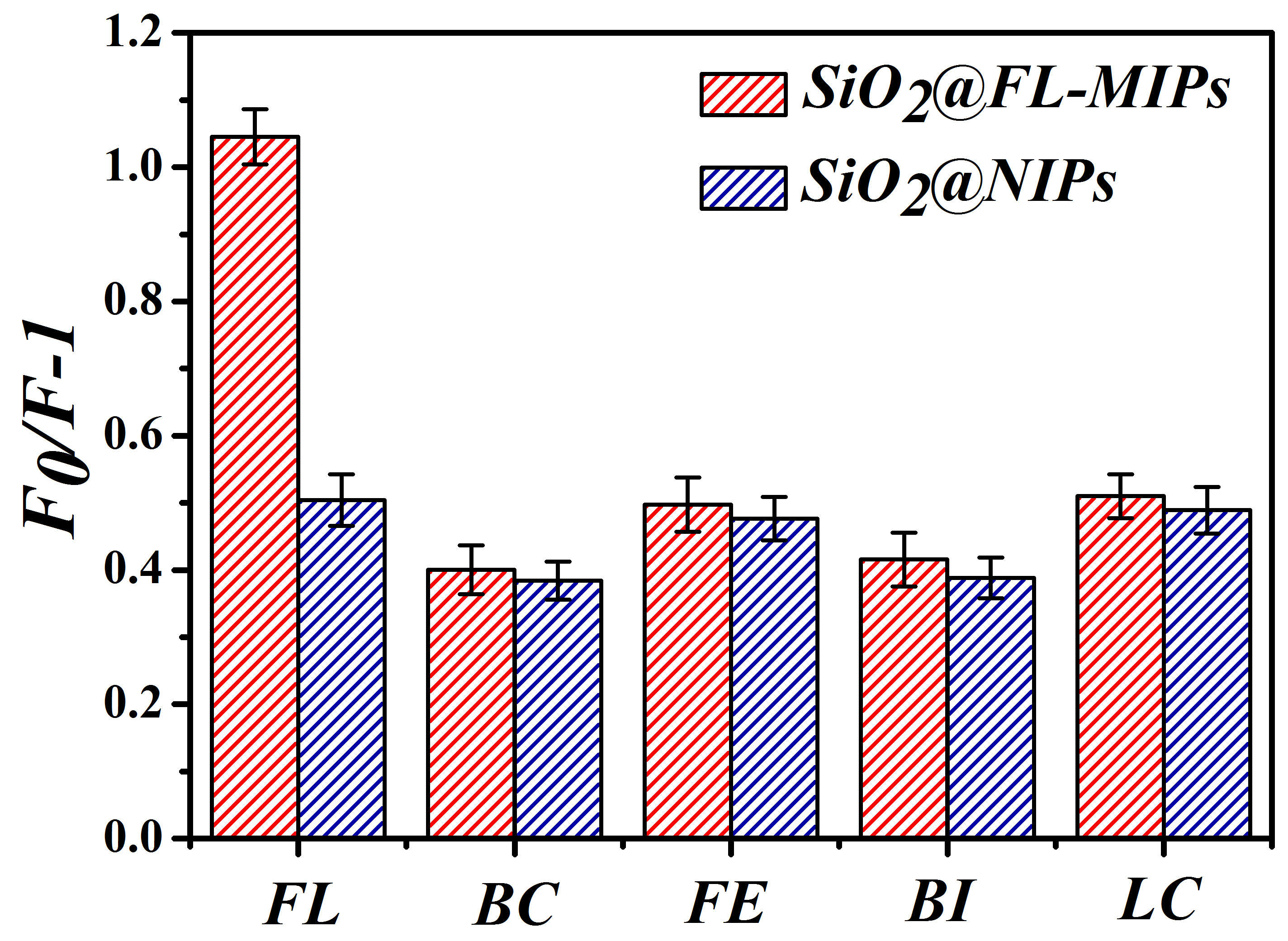


Figure S7. Quenching efficiency of SiO_2_@FL-FMIPs and SiO_2_ @FNIPs by different kinds of 60 nM pyrethroids.


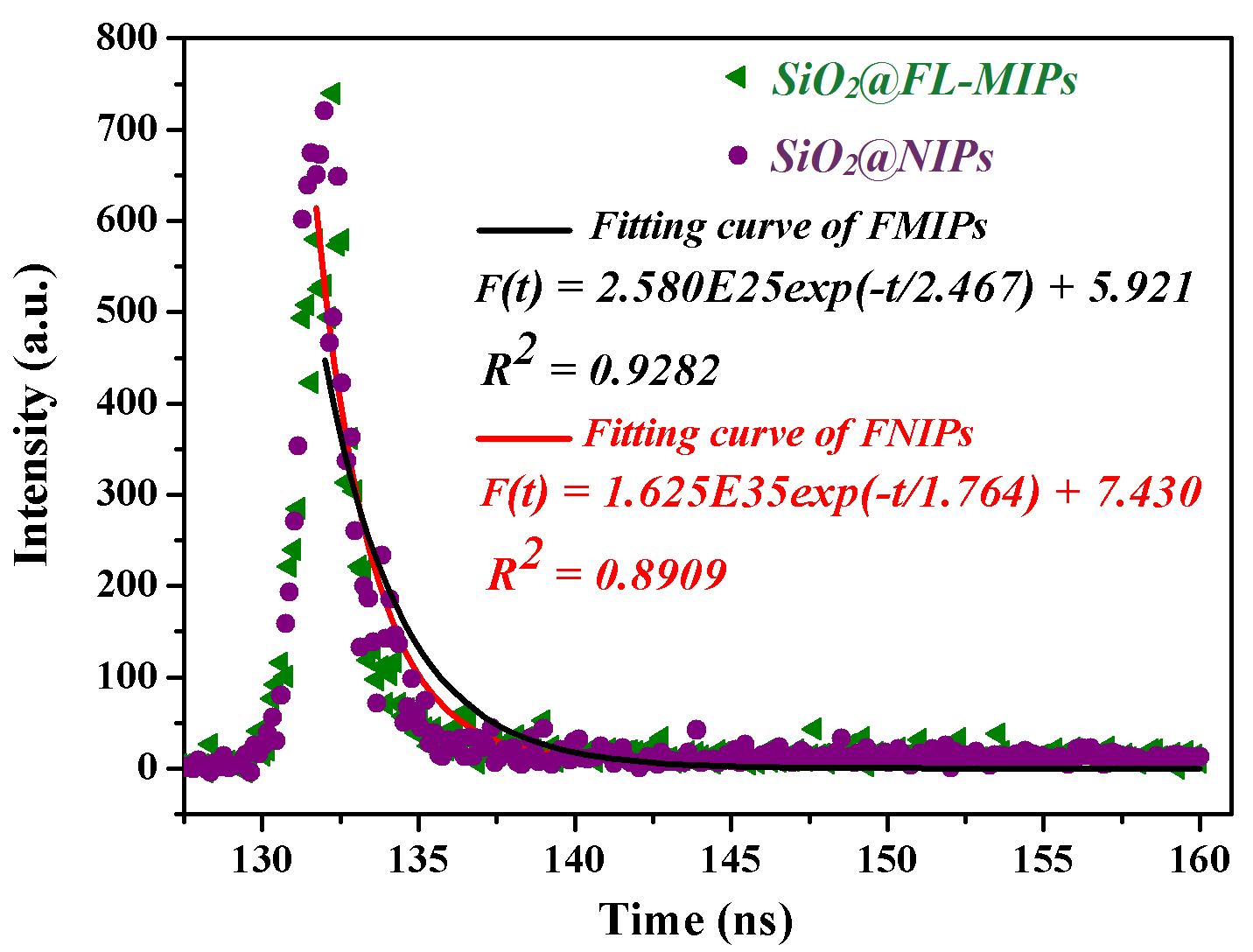


Figure. S8. Transient fluorescence spectra of SiO_2_@FL-FMIPs and SiO_2_@FNIPs, in which the excitation wavelengths are 488 nm. Time-resolved fluorescence curves SiO_2_@FL-FMIPs and SiO_2_@FNIPs are illustrated.


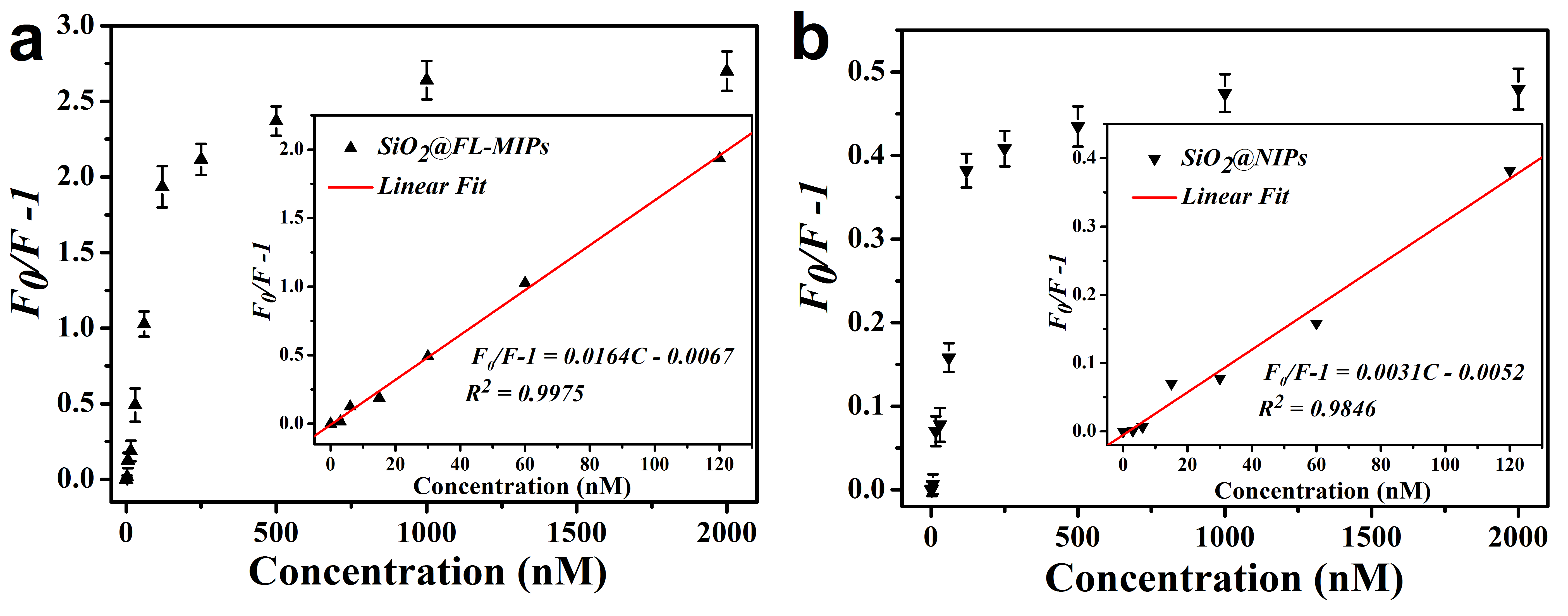


Figure. S9. Fluorescence quenching efficiency changed according to FL concentration. (Insets) Linear equations of SiO_2_@FL-FMIPs (a) and SiO_2_@FNIPs (b).


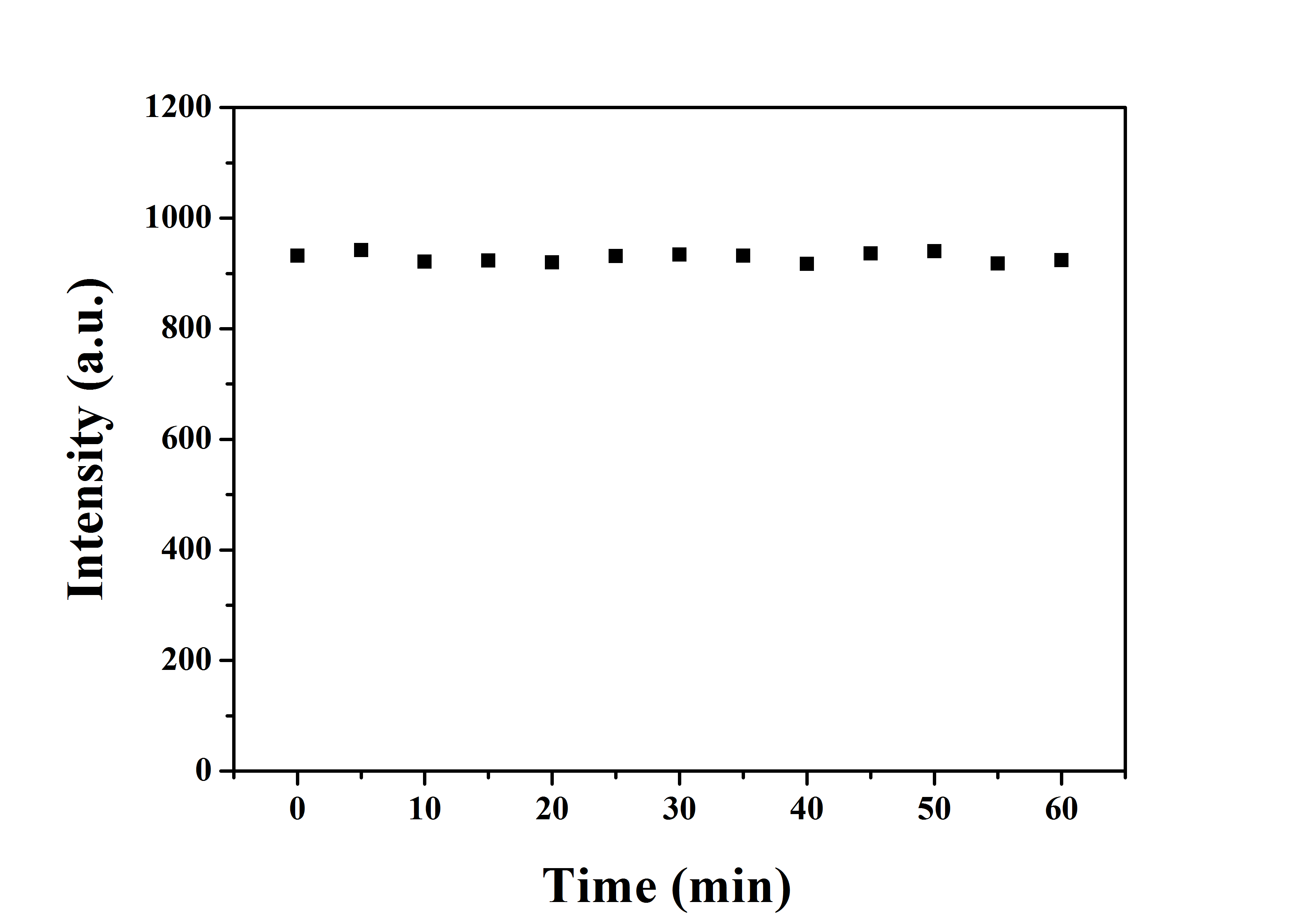


Figure S10. Fluorescence intensity changes of SiO_2_@FL-FMIPs within 60 min.


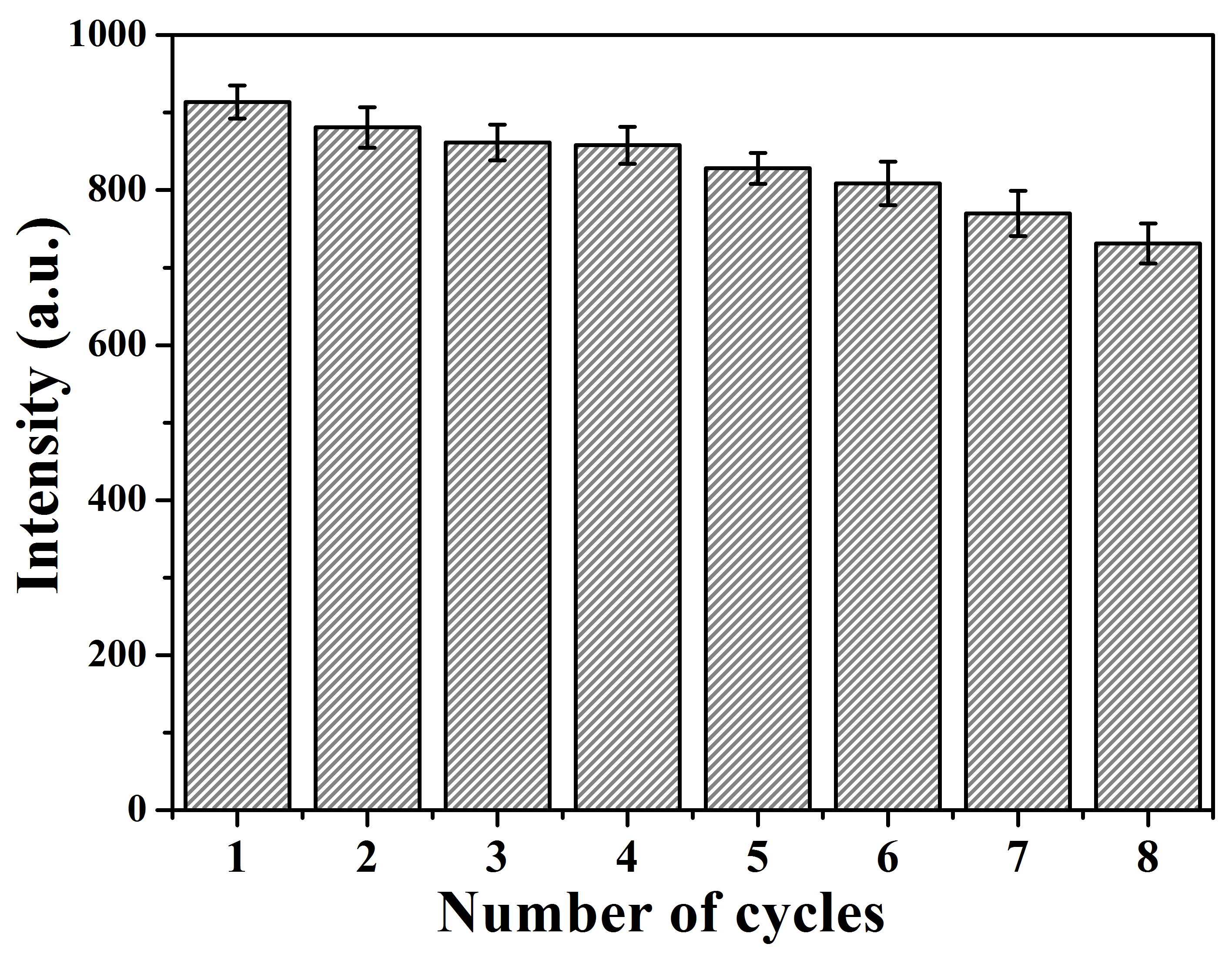


Figure S11. The durability of SiO_2_@FL-FMIPs which was examined through eight binding–desorption cycles. The SiO_2_@FL-FMIPs nanospheres (100 mg) were added into FL solution (60 nM) and incubated for 2 h before fluorescence measurement. After the test, SiO_2_@FL-FMIPs containing FL were washed with methanol-acetic acid solution and rinsed with ethanol, collected by centrifuge in the end. The detection and adsorption procedures were repeated for 8 times by using the same batch of SiO_2_@FL-FMIPs.


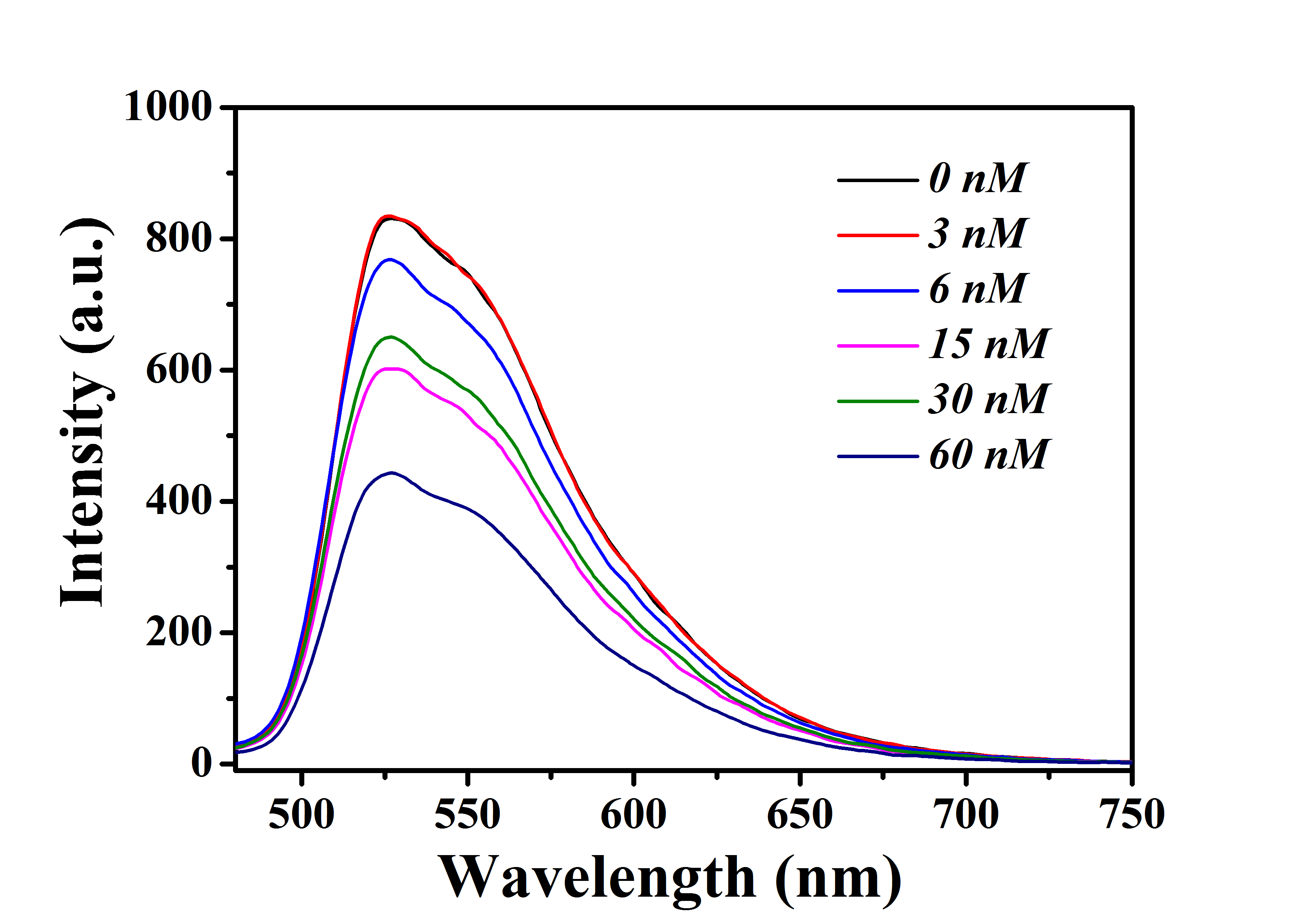


Figure S12. Response curve of SiO_2_@FL-FMIPs to FL in the concentration range of 0-60 nM by seventh recycled materials.


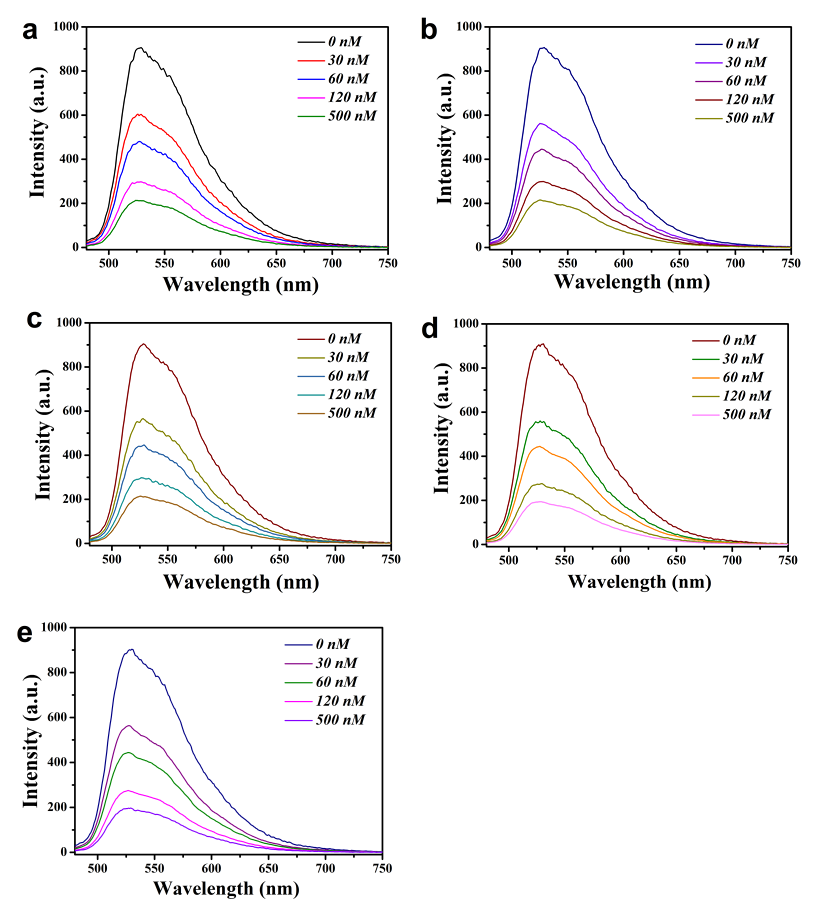


Figure S13. The fluorescence spectra curve of the detection data in Table 1.

**Table S1.** The parameter of transient fluorescence spectrum obtained by using nonlinear fitting

| Samples | Nonlinear equations | Lifetimes (ns) | Correlation coefficients(R^2^) |
| --- | --- | --- | --- |
| SiO_2_@FL-FMIPs | F(t)=2.580E25 exp(-t/2.467)+5.921 | τ=2.467 | 0.9282 |
| SiO_2_@FNIPs | F(t)=1.625E35 exp(-t/1.764)+7.430 | τ_0_=1.764 | 0.8909 |

Table S2 Different methods of detection pyrethroids.

| Methodology | LOD | Linear range | Sample | Ref. |
| --- | --- | --- | --- | --- |
| SiO_2_@FL-FMIPs | 12.145 nmol·L^-1^ | 0-120 nmol·L^-1^ | Taihu lake | This work |
| SPME-HPLC | 0.01 mg·kg^-1^ | 0.05-0.5 mg·kg^-1^ | Cucumber, watermelon | 1 |
| YVO4:Eu^3+^@MIPs | 1.76 μmol·L^-1^ | 2.0-10.0 μmol·L^-1^ | water | 2 |
| SPE-HPLC | 0.02-0.08 μg·L^-1^ | 0.05-25 μg·L^-1^ | water | 3 |
| SDME/GC-FID | 2.74 μg·L^-1^ | 0.3-3.0 μg·L^-1^ | water | 4 |
| SPE | 0.025-0.032 μg·L^-1^ | 0.083-0.106μg·L^-1^ | Tea drinks | 5 |
| GC-ECD | 0.075 mg·kg^-1^ | 0.025-0.05 mg·kg^-1^ | palm oil matrices | 6 |
| MIPs-OVDAC/CdTe QDs | 0.03 μmol·L^-1^ | 0.1-16 μmol·L^-1^ | Yangtze River | 7 |

**Reference**

1 Vazquez, R.P., Mughari, A.R. & Galera, M.M. Solid-phase microextraction (SPME) for the determination of pyrethroids in cucumber and watermelon using liquid chromatography combined with post-column photochemically induced fluorimetry derivatization and fluorescence detection. *Analytica chimica acta* **607**, 74-82 (2008).

2 Liu, C. et al. Molecular Imprinting in Fluorescent Particle Stabilized Pickering Emulsion for Selective and Sensitive Optosensing of lambda-Cyhalothrin. *Journal of Physical Chemistry C* **117**, 10445-10453 (2013).

3 Zhou, Q., Gao, Y., Bai, H. & Xie, G. Preconcentration sensitive determination of pyrethroid insecticides in environmental water samples with solid phase extraction with SiO2 microspheres cartridge prior to high performance liquid chromatography. *Journal of Chromatography A* **1217**, 5021-5025 (2010).

4 Pinheiro, A.d.S. & de Andrade, J.B. Development, validation and application of a SDME/GC-FID methodology for the multiresidue determination of organophosphate and pyrethroid pesticides in water. *Talanta* **79**, 1354-1359 (2009).

5 Wang, Y. et al. Determination of five pyrethroids in tea drinks by dispersive solid phase extraction with polyaniline-coated magnetic particles. Talanta 119, 268-275 (2014).

6 Muhamad, H., Zainudin, B.H. & Abu Bakar, N.K. Comparative study of different clean-up techniques for the determination of lambda-cyhalothrin and cypermethrin in palm oil matrices by gas chromatography with electron capture detection. *Food chemistry* **134**, 2489-2496 (2012).

7 Wei, X. et al. Highly-controllable imprinted polymer nanoshell at the surface of silica nanoparticles based room-temperature phosphorescence probe for detection of 2,4-dichlorophenol. *Analytica chimica acta* **870**, 83-91 (2015).

Table S3. The recovery of FL-containing water samples detected by using HPLC.

| Samples | Test | FL added (nM) | FL detected^*^ (nM) | Recovery (%) |
| --- | --- | --- | --- | --- |
| MiniQ water | 1 | 0 | 0.08 ± 0.02 | - |
|  | 2 | 30 | 31.17 ± 0.75 | 103.89 ± 2.41 |
|  | 3 | 60 | 60.67 ± 1.11 | 101.11 ± 1.83 |
|  | 4 | 120 | 121.67 ± 3.36 | 101.38 ± 2.76 |
|  | 5 | 500 | 506 ± 6.33 | 101.2 ± 1.25 |
| Tap water | 1 | 0 | 0.17 ± 0.04 | - |
|  | 2 | 30 | 31.67 ± 1.44 | 105.56 ± 4.55 |
|  | 3 | 60 | 61.66 ± 2.38 | 102.77 ± 3.86 |
|  | 4 | 120 | 123.21 ± 3.88 | 102.72 ± 3.15 |
|  | 5 | 500 | 508.33 ± 8.78 | 101.67 ± 1.73 |
| Taihu Lake water 1 | 1 | 0 | 0.2 ± 0.06 | - |
|  | 2 | 30 | 32 ± 2.21 | 106.67 ± 6.91 |
|  | 3 | 60 | 62.30 ± 3.56 | 103.83 ± 5.71 |
|  | 4 | 120 | 124.86 ± 5.19 | 104.05 ± 4.16 |
|  | 5 | 500 | 513.65 ± 9.11 | 102.73 ± 1.77 |
| Taihu Lake water 2 | 1 | 0 | 0.23 ± 0.11 | - |
|  | 2 | 30 | 33.17 ± 1.79 | 110.56 ± 5.41 |
|  | 3 | 60 | 62 ± 3.66 | 103.33 ± 5.90 |
|  | 4 | 120 | 127.14 ± 4.78 | 105.92 ± 3.76 |
|  | 5 | 500 | 515 ± 8.66 | 103 ± 1.68 |
| Taihu Lake water 3 | 1 | 0 | 0.16 ± 0.04 | - |
|  | 2 | 30 | 32.4 ± 2.53 | 108.0 ± 7.81 |
|  | 3 | 60 | 62.33 ± 4.21 | 103.88 ± 6.75 |
|  | 4 | 120 | 124.93 ± 4.89 | 104.11 ± 3.91 |
|  | 5 | 500 | 510.32 ± 8.98 | 102.06 ± 1.76 |

^*^ Average of three measurements.

1. ^^ *Corresponding author. Tel.: +86 051188791800*

   *E-mail address: yys@mail.ujs.edu.cn* [↑](#footnote-ref-2)
